# Exploration of UHPLC-ESI-QTOF-MS Profiles and the Neuroprotective, Antidiabetic, Antioxidant and Cytotoxic Effects of Extracts from *Achillea maritima* (L.) Ehrend. & Y.P.Guo (Asteraceae) Collected in Türkiye

**DOI:** 10.1007/s11130-025-01314-x

**Published:** 2025-02-13

**Authors:** Shakeel Ahmed, Gokhan Zengin, Álvaro Fernández-Ochoa, Maria de la Luz Cádiz-Gurrea, Francisco Javier Leyva-Jiménez, Omer Elkiran, Ugur Cakilcioglu, Bengusu H. Akgul, Catarina G. Pereira, Luísa Custódio

**Affiliations:** 1 Department of Biology, Science Faculty, Selcuk University, Konya, 42130 Turkey; 2 Department of Analytical Chemistry, Faculty of Sciences, University of Granada, Fuentenueva s/n, Granada, E- 18071 Spain; 3 REQUIMTE/LAQV, Polytechnic of Porto– School of Engineering, Rua Dr. António Bernardino de Almeida, Porto, 4249-015 Portugal; 4 Department of Environmental Health, Vocational School of Health Services, Sinop University, Sinop, Turkey; 5 Pertek Sakine Genç Vocational School, Munzur University, Pertek, 62500 Turkey; 6 Centre of Marine Sciences (CCMAR/CIMAR LA), Faculty of Sciences and Technology, University of Algarve, Ed. 7, Campus of Gambelas, Faro, 8005-139 Portugal

**Keywords:** *A. maritima*, Enzyme inhibition, Cytotoxicity, Antioxidant, Chemical profiling

## Abstract

The current study investigated the chemical composition, antioxidant activity, enzyme inhibition, and cytotoxic activities of extracts from *Achillea maritima*, a wild medicinal plant used for various therapeutic purposes. The antioxidant activities were assayed through different assays like DPPH, ABTS, CUPRAC, FRAP, and phosphomolybdenum, whereas in enzyme inhibition studies, cholinesterase, tyrosinase, α-amylase, and α-glucosidase were assayed. Cytotoxicity studies are conducted on S17, RAW, and HepG2 to assess its selectivity and effectiveness. Chemical profiling by UHPLC-ESI-QTOF-MS revealed multiple bioactive compounds in the extracts. Polar solvents (ethanol, ethanol/water, and water) resulted in high concentrations of phenolic acids as well as chlorogenic and caffeoylquinic acids, as well as flavonoids like vicenin and apigenin. On the other, the nonpolar (hexane extract) was rich in octadecatrienoic acid hydroperoxy and hydroxyoctadecatrienic acid. Among these, the water extract contained the highest phenolic content of 32.26 mg GAE/g, while the ethyl acetate extract was the richest in flavonoids, with 7.83 mg RE/g. In the antioxidant studies, the water and ethanol/water extracts consistently display the most potent activities, thus indicating their significant free radical scavenging and metal chelation abilities. The studies on enzyme inhibitions showed remarkable BChE inhibitory activities of the ethanol extract in 12.50 mg GALAE/g, thus showing potential in managing disease conditions related to cholinesterase. Tyrosinase inhibition was significant by the ethanol extract, presenting 55.59 mg KAE/g. The ethyl acetate extract exhibited the most potent inhibitory activity against α-amylase with 0.66 mmol ACAE/g, while ethanol extract showed significant inhibition of α-glucosidase with 4.35 ACAE/g. Cytotoxicity results showed that the water extract was most effective against the HepG2 cancer cell line by reducing cell viability to 38.4% at high doses while preserving low toxicity against normal cells, as observed by high viability percentages in S17 and RAW cell lines. These results highlight the usefulness of *A. maritima* extracts in nutraceutical, pharmaceutical, and cosmeceutical applications.

**Supplementary Information:**

The online version contains supplementary material available at 10.1007/s11130-025-01314-x.

## Introduction

Over the last decade, the terms antioxidants and enzyme inhibitors have gained interest in the scientific community. These terms are related to health-promoting applications. Antioxidants represent a powerful defence mechanism against the attack of free radicals and can, therefore, control the progression of global health problems such as cancer, diabetes, and cardiovascular problems [1]. Regarding enzyme inhibitors, inhibiting key enzymes can ease the symptoms of serious health problems such as diabetes, Alzheimer’s, and obesity. With this in mind, some compounds have been chemically produced as antioxidants and enzyme inhibitors. However, most have unpleasant side effects when used for a long time. Therefore, alternative, natural, safe sources of antioxidants and enzyme inhibitors are needed to replace them. Polyphenols are the most prevalent bioactive compounds in plants, vegetables, and fruits, with significant antioxidant effects and nutritional advantages [2, 3]. They are employed in various sectors, including food, pharmaceuticals, and cosmetics. The extraction process employed is crucial in isolating and identifying high-value active components, notably polyphenols, from plants. Consequently, numerous researchers have investigated the impact of various extraction methods on yielding phenolic compounds from plant sources [4, 5]. The polarity of the solvent employed influences the solubility of phenolic compounds. The extraction process, duration, and temperature significantly influence the determination of phenolic chemicals from plant sources. Consequently, it is challenging to devise an extraction procedure that effectively isolates all phenolic chemicals from plants, especially wild plants. Several parameters, including temperature, time, solvent amount, and polarity, influence the extraction and purification of phytochemicals from plant material. Due to their chemical composition, diverse phytochemicals are extracted using polar solvents, as no single solvent can effectively extract all phytochemical and antioxidant components found in plant material. The serial exhaustive extraction method entails the sequential extraction using solvents of ascending polarity, starting from a non-polar solvent (n-hexane) to a more polar solvent (water), to guarantee the extraction of a diverse array of compounds with varying polarities. Research indicates that solvent polarity substantially influences phenolic compounds’ extraction yield and antioxidant efficacy in plant materials [6]. The growing recognition of polyphenols’ commercial potential compels the industry to seek novel, sustainable methods and solvents for their extraction. The flora of Turkey is exceptionally diverse since over 3,000 species of medicinal and aromatic plants are represented [7]. The genus *Achillea (*Asteraceae*)* comprises circa 100 species worldwide, originating from Southwest Asia and Southeast Europe and dispersed over the whole Eurasian subcontinent to North America [8]. The main habitats of *Achillea* species can be found in countries like Iran, Türkiye, Serbia, and parts of Eastern Europe. In Türkiye, there are 48 species and 54 taxa of this genus, of which 24 are endemic [9]. *Achillea* is a well-known medicinal herb; due to its healing properties, its usage in medication has been documented for thousands of years. Various phytochemical studies identified that most of the plants of this genus bear bioactive phytochemicals with unequivocal health benefits [10, 11]. *Achillea maritima* (L.) Ehrend. & Y.P. Guo, a low shrub, is white and hairy on leaves and stems and it grows wild. Plant up to 40–50 cm high, oblong-lanceolate leaves, slightly serrated along margin and subacute at apex, measuring 10–20 × 3–7.5 mm: small hemispherical heads on several stems [11], with white cotton-like scales [12, 13]. As an ethnobotanical application, the infusion of its leaves is used against coughs, menstrual cramps, and haemorrhoids and as an antidiuretic and anthelmintic [14]. Although its essential oil characterization and beneficial properties were previously reported [15], the phytochemical characterization of phenolic and their related bioactivity have been scarcely investigated. In this scenario, the present study aims to evaluate the antioxidant potential, enzyme inhibition, and cytotoxicity of various extracts (hexane, ethyl acetate, ethanol, Ethanol/water (70%), and water infusion) obtained from the aerial parts of *A. maritima*. It also involved the phytochemical analysis with HPLC-ESI-QTOF-MS of the *A. maritima*. The obtained results may open new horizons for potential applications, especially for developing health-promoting applications and functional foods using *A. maritima*. The present study can provide a strong scientific starting point for further applications of the plant in the nutraceutical industry.

## Materials and Methods

The Materials and methods section is presented as Supplementary Material.

## Results and Discussion

The study extracted the plant material with five solvents, and crude extracts were obtained for each solvent. The extraction yields are given in Table 1. The extraction yields depend on the solvents, and the highest extraction yield was obtained with water (11.58%), followed by ethanol/water (9.70%) and ethanol (4.25%). The lowest extraction yield was recorded in the hexane extract with 1.15%.

**Table 1:** Total phenolic and flavonoid contents in the tested extracts

Extracts	Extraction yields (%)	TPC (mg GAE/g)	TFC (mg RE/g)
Hexane	1.15	19.28 ± 0.29^d^	2.02 ± 0.11^e^
Ethyl acetate	1.76	28.81 ± 1.20^b^	7.83 ± 0.10^a^
Ethanol	4.25	25.37 ± 0.06^c^	6.11 ± 0.08^b^
Ethanol/water (70%)	9.70	29.20 ± 0.27^b^	3.41 ± 0.05^c^
Water (infused)	11.58	32.26 ± 0.14^a^	2.83 ± 0.38^d^

^*^Values are reported as mean ± SD of three parallel measurements. GAE: Gallic acid equivalents; RE: Rutin equivalents. Different letters indicate significant differences between the tested extracts (*p* < 0.05)

### Total Phenolic (TPC) and Flavonoid Contents (TFC)

Phenolic compounds are one of the most important classes of secondary metabolites that have great significance for their pharmacological action in humans, such as antioxidants, antidiabetics, and anticancer agents, among many other biological activities. The influence of different extraction solvents on phenolic content and flavonoid contents in the extracts obtained for various parts of *A. maritima* was evaluated in the present work. Table 1 presents the results gathered.

The highest total phenolic contents were in water extract, 32.26 mg GAE/g, followed closely by ethanol/water 70% extract at 29.20 mg GAE/g. Other solvents showing high values of phenolic contents were ethyl acetate and ethanol extracts of 28.81 mg GAE/g and 25.37 mg GAE/g, respectively. The hexane extract exhibited the lowest total phenolic concentration, 19.28 mg GAE/g, indicating that more polar solvents better extract the phenolic components of *A. maritima*. However, these contents were lower than in a previous study by Bilgi et al. [11]. The highest total flavonoid content was obtained for the ethyl acetate extract, 7.83 mg RE/g, which stipulated that the flavonoid principles are highly concentrated in this fraction. The ethanol extract was followed with a high concentration of flavonoids, 6.11 mg RE/g; after that, ethanol/water extract, 3.41 mg RE/g, and water extract, 2.83 mg RE/g. The lowest concentration was found in the hexane extract at 2.02 mg RE/g. This, therefore indicates that flavonoid content is analogous to the phenolic content in being more prevalent in extracts from polar solvents.

### Chemical Characterization

Chemical characterization of *A. maritima* extracts was done to unravel a wide range of bioactive compounds, showing variation in different classes of solvents used in the study. A list of all identified compounds and retention indices in all the extracts is depicted in Table S1-S2 and Figure S1. Hexane, being nonpolar, was dominated by acids like octadecatrienoic acid hydroperoxy and hydroxyoctadecatrienic acid, compounds previously associated with anti-inflammatory activity, even though it has poor antioxidant activity. Polar solvent extracts of ethanol, ethanol/water, and water displayed a complex composition dominated by phenolic acids, among which caffeoylquinic acid isomers predominated; flavonoids such as vicenin isomers; organic acids like quinic and malic acid; and other bioactive compounds are known for their good antioxidant and anti-inflammatory activity. These data indicate that polar extracts of *A. maritima* possess high potential for their application in medicine, especially for preventing diseases connected with oxidative stress. These results align with those from previously reported studies on *A. maritima* [11], in which various bioactive compounds were identified, including caffeic acid, rutin, p-coumaric acid, ferulic acid, rosmarinic acid, resveratrol, luteolin, apigenin, kaempferol, protocatechuic acid, and many others, including epigallocatechin gallate and naringenin. Like this study, ferulic acid was present in many of the solvent extracts in that study; this also further establishes the chemical versatility of this compound in polar solvent systems. Also, protocatechuic acid has been reported as a phenolic acid for its antioxidant and anti-inflammatory activities [16]; thus, this could support the significant contribution of this compound to the bioactivity of *A. maritima*. The congruence in compounds among the same or similar plant species generally points toward the reproducibility of different extraction methods in terms of the bioactive profile for *A. maritima*. Comparing these findings with other species of *Achillea* [17, 18], such as *A. millefolium*,*A. tenuifolia*, and *A.* c*hryschoma* several similarities and differences can be observed. Other *Achillea* species are generally characterized by terpenes such as α-pinene, camphene, and 1,8-cineole, primarily associated with the aromatic properties of these plants [17]. However, using polar solvent extraction, *A. maritima* is particularly enriched in the chemical profile with phenolic acids and flavonoids, not so predominant in other *Achillea* species. This fact may further reflect a difference in therapeutic applications from other species, mainly antioxidant and anti-inflammatory activities. On the other hand, significant constituents identified in the aerial parts of *A. millefolium* included 2-methyl butanal, β-pinene, and 1,8-cineole [17]. The present study placed greater emphasis on phenolic and flavonoid compounds. The current findings confirm *A. maritima*’s therapeutic potential and that polar extracts from this plant might be significantly rich in antioxidant and anti-inflammatory activities, differing from other species of *Achillea*, which seemed more intent upon terpene content. Further research should be focused on the exact mechanism by which such compounds exercise their bioactive property and possible application in medicinal formulations.

### Antioxidant Activities

Oxidative stress is implicated in the pathogenesis of several human diseases, including diabetes, cancer, heart disease, and other disorders. As antioxidants hold the key to preventing or delaying the onset of oxidative stress, they have become the subject of growing interest as culinary preservatives, natural health products, and food supplements. Various studies have reported that plant secondary metabolites, especially essential oils and phenolics, reduce oxidative damage by preventing free radicals from cellular damage. Numerous antioxidant activities that had been investigated in this study enabled us to confirm a linear correlation expressed in many previous publications, concerning the amount and content of phenolic and flavonoid with antioxidant activity.

**Table 2:** Antioxidant properties of the tested extracts (IC_50_ (mg/ml))

Extracts	DPPH	ABTS	CUPRAC	FRAP	Chelating	PBD
Hexane	> 10	6.17 ± 0.71^a^	2.69 ± 0.10^a^	2.44 ± 0.16^a^	2.20 ± 0.34^bc^	1.49 ± 0.04^c^
Ethyl acetate	5.31 ± 0.20^a^	2.86 ± 0.09^b^	1.57 ± 0.03^b^	1.37 ± 0.03^b^	2.54 ± 0.31^b^	0.99 ± 0.06^e^
Ethanol	4.09 ± 0.01^b^	2.61 ± 0.05^c^	1.61 ± 0.01^b^	1.34 ± 0.03^b^	9.29 ± 0.86^a^	1.36 ± 0.02^d^
Ethanol/water (70%)	1.73 ± 0.03^c^	1.62 ± 0.01^d^	1.34 ± 0.01^c^	0.74 ± 0.02^d^	1.40 ± 0.04^c^	1.72 ± 0.01^b^
Water (infused)	1.48 ± 0.03^d^	1.33 ± 0.01^e^	1.39 ± 0.02^c^	0.85 ± 0.01^c^	1.26 ± 0.06^d^	2.15 ± 0.07^a^
Trolox	0.05 ± 0.001^e^	0.08 ± 0.001^f^	0.10 ± 0.001^d^	0.03 ± 0.001^e^	-	0.47 ± 0.03^f^
EDTA	-	-	-	-	0.25 ± 0.01^e^	-

^*^Values are reported as mean ± SD of three parallel measurements. PBD: Phosphomolybdenum; Different letters indicate significant differences between the tested extracts (*p* < 0.05)

The antioxidant properties of plant-based materials should be tested by exploiting different assays, not only to understand the action of different pathways better but also for a more complete analysis of the antioxidant capacity. The antioxidant activities of the *A. maritima* extracts have been evaluated using various assays such as DPPH, ABTS, CUPRAC, FRAP chelating, and phosphomolybdenum assays. Antioxidant activities of the differing extracts were highly variable; a water extract exhibited the highest general antioxidant activity. Among these, the water extract from the DPPH assay had the highest activity with an IC_50_ value of 1.48 mg/ml, while the ethanol/water extract presented 1.73 mg/ml. On the opposite side, the hexane extract showed the lowest DPPH activity (> 10 mg/ml). This tendency was similar to most of the assays performed in this work: the activities of the water and ethanol/water extracts were relatively high (Table 2). In the ABTS assay, the water extract again showed the highest activity with an IC_50_ value of 1.33 mg/ml, followed by ethanol/water extract (IC_50_: 1.62 mg/ml). In comparison, hexane extracts exhibited the least activity (IC_50_: 6.17 mg/ml). In the CUPRAC assay, the water extract showed a highly active (EC_50_: 1.39 mg/ml), followed closely by the ethanol/water extract with an EC_50_ value of 1.34 mg /ml. The hexane extract exhibited the least activity, with an EC_50_ value of 2.69 mg/ml. In addition, the FRAP assay resulted in high antioxidant activities from water and ethanol/water extracts of 0.74 and 0.85 mg/ml, respectively. In contrast, the hexane extract exhibited the poorest activity of 2.44 mg/ml (Table 2). The water extract exhibited the highest chelating activity of IC_50_ value of 1.26 mg/ml, closely followed by ethanol/water extract with 1.40 mg/ml. The phosphomolybdenum assay-PBD turn presented a slightly different trend since the highest activity was shown by the ethyl acetate extract, 0.99 mg/ml, followed by that of ethanol extract, 1.36 mg/ml. In contrast, the aqueous extract showed the least activity (EC_50_: 2.15 mg/ml). Overall, one may point out that the water and ethanol/water extracts exhibited better antioxidant activities in most assays, hence a potential source for potent natural antioxidants (Table 2). Regarding the relationship between total bioactive compounds and antioxidant activity, we found the highest total phenolic contents in the ethanol/water and water extracts. In this sense, except for the phosphomolybdenum assay, these extracts showed the most potent antioxidant abilities in the assays. From this point, we could say that phenolic compounds may significantly contribute to the observed antioxidant properties of *A. maritima.* In agreement with our results, several authors reported a linear correlation between total phenolic content and antioxidant properties [19]. In addition to the total bioactive components, some compounds, including caffeoylquinic acids, caffeic acid, or vicenin isomers, are found only in the ethanol/water and water extracts. Thus, the presence of these compounds may contribute to the observed antioxidant properties of these extracts. Consistent with our findings, some researchers reported that the compounds have significant antioxidant properties [20]. The results obtained regarding antioxidant activity are in concordance with previous studies conducted on *A. maritima*. For DPPH and ABTS assays, both studies mentioned that polar extracts like water, ethanol, and ethyl acetate displayed the highest antioxidant activities. In contrast, non-polar extracts like hexane and chloroform exhibited the lowest activities. Indeed, our results on water and ethanol/water extracts have provided similar trends found in the literature where these extracts have time and again shown better antioxidant potential when compared to hexane and chloroform. This again leads to the idea that polar solvents are more effective in extracting the responsible bioactive compounds for antioxidant activity in *Achillea* species.

### Enzyme Inhibition Assays

Enzyme inhibition assays were performed to evaluate the potential of the *A. maritima* extracts in modulating key enzymes involved in neurological, dermatological, and metabolic functions. The enzymes targeted in this study include acetylcholinesterase (AChE), butyrylcholinesterase (BChE), tyrosinase, α-amylase, and α-glucosidase. Each enzyme plays a critical role in different physiological processes, and their inhibition is often explored for therapeutic interventions.

### AChE and BChE Inhibition

Alzheimer’s disease may be thought of as the most common neurodegenerative malady leading to dementia in senile patients. AChE is the enzyme responsible for the breakdown of acetylcholine within the synaptic cleft and, thus, serves typically to terminate signal transmission across cholinergic synapses. However, BChE exerts a similar function with a broader substrate specificity and has implications for AD’s disease process. Inhibitors of AChE have consequently been widely investigated for neurodegenerative disorders, including Alzheimer’s disease, where an enhancement in cholinergic signaling may afford symptomatic relief. Results are reported here as IC_50_ values (mg/ml) (Table 3). The ethanol/water (70%) and hexane extract displayed moderate AChE inhibition, with an IC_50_ value of 1.12 mg/ml, indicating potential neuroprotective properties. The other extracts, including ethyl acetate, ethanol, and water extracts, did not exhibit significant AChE inhibition, suggesting limited activity toward this enzyme. For BChE, the ethanol extract showed the highest inhibition, with an IC_50_ value of 1.40 mg/ml, reflecting its strong potential for managing cholinesterase-related disorders. The ethyl acetate extract followed with 2.37 mg/ml, while the hexane extract demonstrated moderate inhibition (IC_50_: 2.59 mg/ml). The water extract did not show BChE inhibition. Based on Table S1, some compounds in the extracts can explain the observed cholinesterase inhibitory effects. In particular, caffeoylquinic acid derivatives and some flavonoids (vicenin, luteolin, eriodictoyl, eupatilin, etc.) can support the cholinesterase inhibitory effect. For example, Grzelczyk et al. [21] reported that 3-O-caffeoylquinic acid significantly inhibited AChE. In another study by Trendifolia et al. [22], the levels of several caffeoylquinic acid derivatives were correlated with the observed AChE inhibitory effect. Vicenin was also reported to inhibit cholinesterase in a previous study reversibly [23]. Additionally, Choi et al. [24] found that luteolin showed a moderate inhibitory effect on AChE.

**Table 3:** Enzyme inhibitory properties of the tested extracts

Extracts	AChE	BChE	Tyrosinase	Amylase	Glucosidase
Hexane	1.12 ± 0.01^a^	2.59 ± 0.18^b^	na	2.14 ± 0.01^c^	1.41 ± 0.01^b^
Ethyl acetate	na	2.37 ± 0.53^b^	na	1.87 ± 0.06^d^	1.29 ± 0.01^bc^
Ethanol	na	1.40 ± 0.23^c^	2.39 ± 0.25^b^	3.29 ± 0.13^b^	1.03 ± 0.01^c^
Ethanol/water (70%)	1.12 ± 0.02^a^	6.05 ± 1.38^a^	9.19 ± 1.26^a^	4.53 ± 0.03^a^	1.15 ± 0.26^bc^
Water (infused)	na	na	na	> 10	> 10
Galantamine	0.003 ± 0.0001^b^	0.001 ± 0.0001^d^	-	-	-
Kojic acid	-	-	0.13 ± 0.01^c^	-	-
Acarbose	-	-	-	0.80 ± 0.01^e^	2.90 ± 0.14^a^

^**^Values are reported as mean ± SD of three parallel measurements. na: not active. Different letters indicate significant differences between the tested extracts (*p* < 0.05)

### Tyrosinase Inhibition

The anti-tyrosinase assay is based on the inhibition of tyrosinase obtained from mushrooms. Tyrosinase is a well-known enzyme for its key role in melanin biosynthesis and dermatological disorders such as melanoma, age spots, and freckles due to excessive accumulation of melanin. Thus, tyrosinase inhibitors have been of greater interest in treating skin disorders. In our experiments, only ethanol and ethanol/water extracts of *A. maritima s*howed tyrosinase inhibitory activity. The inhibition results are expressed IC_50_ values (Table 3). The ethanol extract exhibited the most potent tyrosinase inhibition, with an IC_50_ value of 2.39 mg/ml, highlighting its potential use in dermatological treatments and cosmetic formulations. The ethanol/water extract also showed weak inhibition (IC_50_: 9.19 mg/ml). Neither the hexane nor the ethyl acetate extracts demonstrated significant tyrosinase inhibitory activity. No previous study on tyrosinase inhibition of A. maritima is available in the literature. At the same time, many other species of *Achillea* represent significant findings on inhibiting this important enzyme that corresponds to the current results of *A. maritima* [25–28]. In previous studies on some members of the genus *Achiella*, the tyrosinase inhibitory effect may be attributed to the presence of some compounds, particularly caffeoylquinic acid derivatives. For example, Strzpek-Gomka et al. [26] investigated the tyrosinase inhibitory effect of *A. biebersteinii* and reported the relationship between the presence of caffeoylquinic acid derivatives and the tyrosinase inhibitory effect. In another study by Kim et al. [29], 5-caffeoylquinic acid was an effective anti-melanogenesis agent. In addition to phenolic acids, some flavonoids in the tested extracts can contribute to the observed tyrosinase inhibitory effects. For example, the inhibitory effect of luteolin on tyrosinase was reported by Zhang et al. [30], and the presence of hydroxyl groups in the B ring contributes to the inhibitory effect. Imen et al. [31] also reported that eriodictyol strongly inhibited tyrosinase in in vivo and in vitro tests.

### α-Amylase and α-Glucosidase Inhibition

α-Amylase and α-glucosidase are classified under enzymes that catalyze the hydrolysis of carbohydrates. The enzyme α-amylase catalyzes an initial step of carbohydrate digestion, being the breakdown of starch into oligosaccharides, while α-glucosidase further cleaves these oligosaccharides into monosaccharides, enabling their absorption as glucose. Inhibition of these enzymes is a widely used principle in controlling postprandial hyperglycemia that characterizes type 2 diabetes. The different extracts of *A. maritima* were investigated for their potential anti-diabetic effect for the first time. The results are presented as IC_50_ values (mg/ml) (Table 3). For α-amylase, the ethyl acetate extract exhibited the highest inhibitory activity, with an IC_50_ value of 1.87 mg/ml, followed by the hexane extract (IC_50_: 2.14 mg/ml). The ethanol extract showed moderate inhibition (IC_50_: 3.29 mg/ml), while the ethanol/water extract and water extract had minimal activity (IC_50_: 4.53 mg/ml and > 10 mg/ml, respectively). Regarding α-glucosidase, the ethanol extract demonstrated the most potent inhibition, with an IC_50_ value of 1.03 mg/ml. The ethanol/water extract recorded moderate inhibition (IC_50_: 1.15 mg/ml). The ethyl acetate extracts also showed significant inhibition (IC_50_: 1.29 mg/ml), followed by the hexane extract (IC_50_: 1.41 mg/ml). However, the water extract exhibited the lowest activity (> 10 mg/ml). Except for water extract, other extracts exhibited stronger glucosidase inhibitory effects compared to acarbose (IC_50_: 2.90 mg/ml). As a realization of the structure-capability relationship, the observed amylase and glucosidase inhibitory effects could be related to the presence of some compounds. For example, Narita and Inouye [32] studied the amylase inhibitory effects of some caffeoylquinic acid derivatives, and the formation of ester bonds in caffeoylquinic acid enhanced their amylase inhibitory effects. Oboh et al. [33] reported significant amylase and glucosidase inhibitory effects of chlorogenic and caffeic acids. In addition to caffeoylquinic acid, vicenin-2 showed a more substantial glucosidase inhibitory effect than acarbose in another study by Islam et al. [34]. The study is the first to pursue research on the antidiabetic activity of this plant species. However, the findings agreed with those from studies on other Achillea species that have been variously reported for their possible antidiabetic agents. Antidiabetic activity that is aimed at reducing blood glucose levels in humans, improving insulin sensitivity, and antioxidant and anti-inflammatory activities have been reported for some species of the genus, including *A. cucullata* [35]. These species have been widely studied for their therapeutic effects against diabetes. Our study revealed similarities with these previous species, weighing on the potential of Achillea species, including this investigated plant, as a promising candidate for further exploration in the search for natural treatments against diabetes. The diversities of bioactive compounds evidenced the shared mechanism of action in species. They strengthened the importance of *Achillea* as a promising genus in the fight against diabetes and related metabolic disorders.

### Cytotoxicity

Different extracts of *A. maritima* were evaluated for cytotoxicity against three mammalian cell lines, namely S17, RAW, and HepG2, each at two different concentrations: 100 µg/mL and 50 µg/mL. The cell lines generally showed low cytotoxicity, but there was marked variation among the extracts. In the case of the normal S17 cell line, all the extracts exhibited high cellular viability, the percentage values exceeding 85% for both concentrations, thus reflecting a minimum cytotoxic effect. Among these, hexane extract showed the highest cell viability at 50 µg/mL, reaching 103.1%, thereby reflecting no significant cytotoxicity toward normal S17 cells. A high viability of the RAW cell line when most of the extracts were applied confirms the low cytotoxicity. At a 50 µg/mL concentration, the highest viability percentage was achieved with both the ethyl acetate and water extracts, reaching 98.3 and 98.6%, respectively. This may suggest that the extracts preserve a minimal effect on RAW cells, representing a good level of selectivity and reducing unspecific cytotoxicity (Table 4). However, although most of the extracts exhibited relatively higher cytotoxicity against the HepG2 cancer cell line, the water extract showed the highest cytotoxicity, especially at the higher concentration of 100 µg/mL, which reduced the viability of the cells to 38.4%. In contrast, treatment with the hexane extract, even at its highest concentration of 100 µg/mL, resulted in higher cell viability of 60.7%, thus showing relatively lower cytotoxic potential against this cancer cell line. This finding also agrees with the previous study on the same plant species, where both the ethanol and chloroform extracts showed potent cytotoxicity against the cancer cell lines. In contrast, water extracts exerted selective cytotoxicity against HepG2 cells. Both studies’ findings exhibited a minimal cytotoxic effect on normal cells, indicating selective activity against cancer cells [11]. These findings emphasize its selective cytotoxicity, with the water extract being strongly active against tumour cells while sparing the normal cells. This increased cytotoxic activity against HepG2 with very minimal activity on S17 normal cell line therefore making the aqueous extract a potential candidate for further studies into its possible applications as an anticancer agent.

**Table 4:** Cellular viability (%) of the extracts on mammalian RAW. HepG2 and S17 cell lines. Applied at 2 concentrations (100 and 50 µg/mL)

Cell line	S17 cells		RAW cells		HepG2 cells	
**Extract**	**100** µg/mL	**50** µg/mL	**100** µg/mL	**50** µg/mL	**100** µg/mL	**50** µg/mL
Hexane	97.7 ± 4.3	103.1 ± 6.4	76.8 ± 7.2	91.6 ± 8.2	60.7 ± 4.2	84.6 ± 7.0
Ethyl acetate	97.5 ± 8.4	102.5 ± 7.4	80.6 ± 5.2	98.3 ± 3.8	59.0 ± 5.1	81.5 ± 6.6
Ethanol	88.9 ± 8.3	90.9 ± 7.4	69.2 ± 1.9	96.9 ± 4.9	76.1 ± 9.1	90.1 ± 8.3
Ethanol/Water	85.6 ± 7.0	88.2 ± 5.9	69.3 ± 5.4	102.8 ± 4.6	49.7 ± 4.5	86.9 ± 7.9
Water	66.7 ± 2.4	88.4 ± 9.2	80.9 ± 6.3	98.6 ± 3.7	38.4 ± 3.5	92.2 ± 8.0

Values represent the mean ± SD (*n* = 6)

## Conclusion

The present work on the chemical composition showed that *A. maritima* from Turkey could be a good source of various natural compounds. The results of antioxidant potential and enzyme inhibition assays develop extracts of *A. maritima* as part of several therapeutic uses. Ethanol extract may possess high tyrosinase and α-glucosidase inhibition activities that could be valuable in dermatological and metabolic health interventions. All the extracts exhibited significant activity regarding the inhibition of α-amylase and α-glucosidase, which justifies their use in carbohydrate digestion management. The hexane extracts also showed a moderate inhibition of several enzymes, such as AChE, BChE, and α-amylase. These results prove that selecting the appropriate solvent is crucial for the extraction of bioactive compounds bearing specific properties of enzyme inhibition. The obtained data underline the potential of *A. maritima* as a source of natural antioxidants and enzyme inhibitors for use in health-promoting applications.

## Electronic Supplementary Material

Below is the link to the electronic supplementary material.

Supplementary Material 1

## Data Availability

Data is provided within the manuscript or supplementary information files.
