# Supplementary material for: Exploration of UHPLC-ESI-QTOF-MS Profiles and the Neuroprotective, Antidiabetic, Antioxidant and Cytotoxic Effects of Extracts from Achillea maritima (L.) Ehrend. & Y.P.Guo (Asteraceae) Collected in Türkiye
Source: Plant Foods Hum Nutr. 2025 Feb 13;80(1):66. doi: 10.1007/s11130-025-01314-x (PMC11825534; doi:10.1007/s11130-025-01314-x)
Supplement: Supplementary file 1 — Supplementary Material 1 [file 11130_2025_1314_MOESM1_ESM.docx]

**Materials and Methods**.

**Plant Collection**

In 2022, botanical specimens were collected from the Akliman area in Sinop, Turkey. Dr. Omer Elkiran conducted the taxonomic identification, and a voucher specimen was preserved in the herbarium of Sinop University (Voucher number: OE-22-30). The aerial parts were separated, dried in the shade at ambient temperature, pulverized, and stored away from light.

**Plant Extract Preparation**

The extraction procedure included five solvents: hexane, ethyl acetate, ethanol, a 70% ethanol/water mixture, and water. Each 10 g sample was macerated with 200 mL of ethyl acetate, ethanol, and a mixture of ethanol and water for 24 hours at ambient temperature. The aqueous extract was prepared by infusing 10 g of plant material in boiling water for 15 minutes. Organic solvents were removed via evaporation under low pressure, and the aqueous extract was subjected to freeze-drying.

**Assays for Total Phenolic and Flavonoid Contents**

The total phenolic content was determined by employing the methods given in the literature with some modiﬁcation. Sample solution (0.25 mL) was mixed with diluted Folin–Ciocalteu reagent (1 mL, 1:9, v/v) and shaken vigorously. After 3 min, Na2CO3 solution (0.75 mL, 1%) was added and the sample absorbance was read at 760 nm after a 2 h incubation at room temperature. The total phenolic content was expressed as milligrams of gallic acid equivalents (mg GAE/g extract)

The total ﬂavonoid content was determined using the AlCl3 method. Brieﬂy, sample solution (1 mL) was mixed with the same volume of aluminum trichloride (2%) in methanol. Similarly, a blank was prepared by adding sample solution (1 mL) to methanol (1 mL) without AlCl3. The sample and blank absorbances were read at 415 nm after a 10 min incubation at room temperature. The absorbance of the blank was subtracted from that of the sample. Rutin was used as a reference standard and the total ﬂavonoid content was expressed as milligrams of rutin equivalents (mg RE/g extract)

**Determination of Antioxidant and Enzyme Inhibitory Effects**

Antioxidant (DPPH and ABTS radical scavenging, reducing power (CUPRAC and FRAP), phosphomolybdenum and metal chelating (ferrozine method)) and enzyme inhibitory activities (cholinesterase (Elmann’s method), tyrosinase (dopachrome method), α-amylase (iodine/potassium iodide method), α -glucosidase (chromogenic PNPG method) and pancreatic lipase (p-nitrophenyl butyrate (p-NPB) method) were determined.

For the DPPH (1,1-diphenyl-2-picrylhydrazyl) radical scavenging assay: Sample solution was added to 4 mL of a 0.004% methanol solution of DPPH. The sample absorbance was read at 517 nm after a 30 min incubation at room temperature in the dark. DPPH radical scavenging activity was expressed as IC_50_ value (mg/ml)

For ABTS (2,2′-azino-bis(3-ethylbenzothiazoline) 6-sulfonic acid) radical scavenging assay: Brieﬂy, ABTS+ was produced directly by reacting 7 mM ABTS solution with 2.45 mM potassium persulfate and allowing the mixture to stand for 12–16 in the dark at room temperature. Prior to beginning the assay, ABTS solution was diluted with methanol to an absorbance of 0.700 ± 0.02 at 734 nm. Sample solution was added to ABTS solution (2 mL) and mixed. The sample absorbance was read at 734 nm after a 30 min incubation at room temperature. The ABTS radical scavenging activity was expressed as IC_50_ value (mg/ml)

For CUPRAC (cupric ion reducing activity) activity assay: Sample solution was added to premixed reaction mixture containing CuCl2 (1 mL, 10 mM), neocuproine (1 mL, 7.5 mM) and NH4Ac buffer (1 mL, 1 M, pH 7.0). Similarly, a blank was prepared by adding sample solution (0.5 mL) to premixed reaction mixture (3 mL) without CuCl2 . Then, the sample and blank absorbances were read at 450 nm after a 30 min incubation at room temperature. The absorbance of the blank was subtracted from that of the sample. CUPRAC activity was expressed as EC_50_ value (effective concentration at absorbace 0.5) (mg/ml)

For FRAP (ferric reducing antioxidant power) activity assay: Sample solution was added to premixed FRAP reagent (2 mL) containing acetate buffer (0.3 M, pH 3.6), 2,4,6-tris(2-pyridyl)-S-triazine (TPTZ) (10 mM) in 40 mM HCl and ferric chloride (20 mM) in a ratio of 10:1:1 (v/v/v). Then, the sample absorbance was read at 593 nm after a 30 min incubation at room temperature. FRAP activity was expressed as as EC_50_ value (mg/ml)

For phosphomolybdenum method: Sample solution was combined with 3 mL of reagent solution (0.6 M sulfuric acid, 28 mM sodium phosphate and 4 mM ammonium molybdate). The sample absorbance was read at 695 nm after a 90 min incubation at 95 °C. The total antioxidant capacity was expressed as EC_50_ value (mg/ml).

For metal chelating activity assay: Brieﬂy, sample solution was added to FeCl2 solution (0.05 mL, 2 mM). The reaction was initiated by the addition of 5 mM ferrozine (0.2 mL). Similarly, a blank was prepared by adding sample solution (2 mL) to FeCl2 solution (0.05 mL, 2 mM) and water (0.2 mL) without ferrozine. Then, the sample and blank absorbances were read at 562 nm after 10 min incubation at room temperature. The absorbance of the blank was subtracted from that of the sample. The metal chelating activity was expressed as as IC_50_ value (mg/ml).

For Cholinesterase (ChE) inhibitory activity assay: Sample solution (was mixed with DTNB (5,5-dithio-bis(2-nitrobenzoic) acid, Sigma, St. Louis, MO, USA) (125 µL) and AChE (acetylcholinesterase (Electric ell acetylcholinesterase, Type-VI-S, EC 3.1.1.7,Sigma)), or BChE (butyrylcholinesterase (horse serum butyrylcholinesterase, EC 3.1.1.8, Sigma)) solution (25 μL) in Tris–HCl buffer (pH 8.0) in a 96-well microplate and incubated for 15 min at 25 °C. The reaction was then initiated with the addition of acetylthiocholine iodide (ATCI, Sigma) or butyrylthiocholine chloride (BTCl, Sigma) (25 μL). Similarly, a blank was prepared by adding sample solution to all reaction reagents without enzyme (AChE or BChE) solution. The sample and blank absorbances were read at 405 nm after 10 min incubation at 25 °C. The absorbance of the blank was subtracted from that of the sample and the cholinesterase inhibitory activity was expressed as as IC_50_ value (mg/ml).

For Tyrosinase inhibitory activity assay: Sample solution was mixed with tyrosinase solution (40 μL, Sigma) and phosphate buffer (100 μL, pH 6.8) in a 96-well microplate and incubated for 15 min at 25 °C. The reaction was then initiated with the addition of L-DOPA (40 μL, Sigma). Similarly, a blank was prepared by adding sample solution to all reaction reagents without enzyme (tyrosinase) solution. The sample and blank absorbances were read at 492 nm after a 10 min incubation at 25 °C. The absorbance of the blank was subtracted from that of the sample and the tyrosinase inhibitory activity was expressed as IC_50_ value (mg/ml).

For α-amylase inhibitory activity assay: Sample solution was mixed with α-amylase solution (ex-porcine pancreas, EC 3.2.1.1, Sigma) (50 μL) in phosphate buffer (pH 6.9 with 6 mM sodium chloride) in a 96-well microplate and incubated for 10 min at 37 °C. After pre-incubation, the reaction was initiated with the addition of starch solution (50 μL, 0.05%). Similarly, a blank was prepared by adding sample solution to all reaction reagents without enzyme (α-amylase) solution. The reaction mixture was incubated 10 min at 37 °C. The reaction was then stopped with the addition of HCl (25 μL, 1 M). This was followed by addition of the iodine-potassium iodide solution (100 μL). The sample and blank absorbances were read at 630 nm. The absorbance of the blank was subtracted from that of the sample and the α-amylase inhibitory activity was expressed as IC_50_ value (mg/ml).

For α-glucosidase inhibitory activity assay: Sample solution was mixed with glutathione (50 µL), α-glucosidase solution (from Saccharomyces cerevisiae, EC 3.2.1.20, Sigma) (50 µL) in phosphate buffer (pH 6.8) and PNPG (4-N-trophenyl-α-D-glucopyranoside, Sigma) (50 µL) in a 96-well microplate and incubated for 15 min at 37 °C. Similarly, a blank was prepared by adding sample solution to all reaction reagents without enzyme (α-glucosidase) solution. The reaction was then stopped with the addition of sodium carbonate (50 µL, 0.2 M). The sample and blank absorbances were read at 400 nm. The absorbance of the blank was subtracted from that of the sample and the α-glucosidase inhibitory activity was expressed as IC_50_ value (mg/ml).

**UHPLC-ESI-QTOF-MS Profiling and Data Processing.**

The extracts obtained from *A. maritima* were prepared at a concentration of 5000 µg/mL in the same solvent applied during extraction and then filtered through 0.22µm. The analyses were carried out using an ACQUITY UHPLC H-Class System (Waters Corp.) coupled to a QTOF-MS (Synapt G2, Waters Corp.) with a reversed-phase column (ACQUITY UHPLC BEH SHIELD RP18, 130 Å, 1.7 μm, 2.1mm× 150 mm) at 22°C. The multi-step gradient used to separate the phytochemicals in each of the extracts was 0.00 min, 99% A; 2.33 min, 99% A; 4.37 min, 93% A; 8.11 min, 86% A; 12.19 min, 76% A; 15.99 min, 60% A; 18.31 min, 2% A; 21.03 min, 2% A; 22.39 min, 99% A and 25.00, 99% A.; where phase A was water acidified with acetic acid (0.5% v/v) and phase B Acetonitrile. Finally, the initial conditions were maintained for 5 minutes to prepare the equipment for the following analyses.

The source parameters of the mass spectrometer coupled to UHPLC instrument were as follows: m/z range 50-1200 m/z; capillary voltage, 2.2 kV; cone voltage, 30 V; desolvation temperature, 500 ℃; desolvation gas glow, 700 L/h; cone gas flow, 50 L/h; source temperature, 100℃; scan duration 0.1s; resolution, 20000 full width at half maximum; ionization mode, negative. The acquisition was performed using two parallel scan functions: low collision energy (4 eV) and elevated collision energy (MSE energy linear ramp: 20 to 60 eV).

On the other hand, the physicochemical characterization of the raw data obtained by the analytical platform used was performed using the open-source software MZmine V4.08. First, the raw data were converted to mzML format using the MSConvertGUI software (v3.0.24). Subsequently, the noise level was set to an intensity of 2E3, and the base-peak chromatograms were obtained by an automated data analysis pipeline (ADAP), setting the m/z tolerance at 15ppm and a minimum highest intensity at 1E4. The chromatograms were subsequently deconvoluted using the local minimum resolver algorithm, setting the following parameters: S/N threshold: 50; minimum feature height: 1E4; coefficient/area threshold: 110; peak duration range: 0.05-0.3 min; RT wavelet range: 0-0.05. Moreover, an isotope grouper algorithm was also applied, and the obtained features were aligned using a m/z tolerance of 15ppm and an RT tolerance of 0.25 min. Finally, the phytochemical identification of the obtained features was determined using Sirius software (v5.8.6) and comparing the obtained molecular formulas and MS/MS spectra in different databases (MoNA, HMDB, SciFinder…).

**Cell culture**

Human hepatocarcinoma cell line (HepG2) was kindly provided by the Center for Biomedical Research (CBMR), University of Algarve, Portugal, while the murine leukemic macrophages (RAW264.7), were purchased from CLS Cell Line Service GmbH, Germany. The HEK 293 cell line (human embryonic kidney) were from CCMAR. All cells were cultured in Dulbecco’s Modified Eagle Medium (DMEM) containing 10% fetal bovine serum, 2 mM L-glutamine (1%), and antibiotics (50 U/mL penicillin and 50 μg/mL streptomycin) (1%). The cultures were maintained in an humidified atmosphere at 37°C with 5% CO_₂._

**Determination of Cellular Viability**

Exponentially growing HepG2 cells (5×10³ cells/well), and RAW 264.7 and HEK 293 cells (1×10⁴ cells/well) were plated in 96-well plates and incubated overnight. Cells were then treated with the extracts at a concentration of 100 μg/mL for 72 hours, with 0.5% DMSO used as a control. Cellular viability was determined via the MTT assay (3-(4,5-dimethylthiazol-2-yl)-2,5-diphenyltetrazolium bromide). In brief, two hours before the incubation period concluded, 20 µL of MTT solution (5 mg/mL in PBS) was added to each well and incubated at 37 °C. Formazan crystals were then dissolved by adding 150 µL of DMSO to each well. Absorbance was measured at 595 nm using an EZ Read 400 microplate reader (Biochrom, Cambridge, UK), and cell viability was calculated as a percentage relative to a 0.5% DMSO control.

**Statistical Analysis**

The experiments were executed in triplicate, and differences among the extracts were assessed using an ANOVA and Tukey's test (p<0.05). The statistical analysis was conducted using Graph Pad Prism (version 9.0).

Table S1. Chemical characterization of the tested extracts

| **Experimental m/z** | **Retention time (min)** | **Adduct** | **Molecular Formula** | **Proposed compound** | **MS/MS Fragments** |
| --- | --- | --- | --- | --- | --- |
| *Organic acids* | | | | | |
| 225 .0628 | 0 .92 | [M-H]^-^ | C_7_H_14_O_8_ | Hexa hydroxyheptanoic acid | 59/71/89/113/119/179 |
| 195 .0525 | 0 .98 | [M-H]^-^ | C_6_H_12_O_7_ | Gluconic acid | 59/75/99/129/159/177 |
| 191 .0569 | 1 .02 | [M-H]^-^ | C_7_H_12_O_6_ | Quinic acid | 59/72/85/99/129/148 |
| 133 .0153 | 1 .08 | [M-H]^-^ | C_4_H_6_O_5_ | Malic acid | 71/73/89/115 |
| 191 .0215 | 1 .19 | [M-H]^-^ | C_6_H_8_O_7_ | Citric acid | 57/87/111/129 |
| 173 .0080 | 1 .81 | [M-H]^-^ | C_6_H_6_O_6_ | Aconitic acid | 59/67/85/ |
| 175 .0605 | 3 .63 | [M-H]^-^ | C_7_H_12_O_5_ | Isopropylmaleate isomer 1 | 59/85/113/115 |
| 157 .050 | 5 .31 | [M-H]^-^ | C_7_H_10_O_4_ | Isopropylmaleate isomer 2 | 113 |
| *Aminoacids and derivatives* | | | | | |
| 293 .1001 | 0 .91 | [M-H]^-^ | C_10_H_18_N_2_O_8_ | Glycosyl-asparagine | 70/114/131/173/203 |
| 203 .0817 | 2 .52 | [M-H]^-^ | C_11_H_12_N_2_O_2_ | Tryptophan | 74/116/142/159 |
| *Sugars and Derivatives* | | | | | |
| 341 .1103 | 0 .96 | [M-H]^-^ | C_12_H_22_O_11_ | Sucrose | 165/179/297 |
| 179 .0570 | 0 .98 | [M-H]^-^ | C_6_H_12_O_6_ | Fructose | 59/71/89 |
| 165 .0419 | 1 .01 | [M-H]^-^ | C_5_H_10_O_6_ | Xylonic acid | 59/87/105/129/147 |
| 267 .0734 | 1 .02 | [M+CH_2_O_2_-H]^-^ | C_8_H_14_O_7_ | Acetyl glucose | 51/71/101/113/131 |
| 209 .0327 | 1 .03 | [M-H]^-^ | C_7_H_14_O_7_ | Heptose | 57/71/85/129/159 |
| 311 .0979 | 1 .19 | [M-H]^-^ | C_11_H_20_O_10_ | Saccharide derivate |  |
| 203 .0212 | 1 .30 | [M-H]^-^ | C_7_H_8_O_7_ | Daucic acid | 57/71/79/97 |
| 311 .0980 | 1 .40 | [M-H]^-^ | C_11_H_20_O_10_ | Saccharide derivate | 59/163/205/267 |
| 281 .0874 | 1 .64 | [M-H]^-^ | C_10_H_18_O_9_ | Xylobiose | 59 |
| 431 .1909 | 6 .66 | [M+CH_2_O_2_-H]^-^ | C_19_H_30_O_8_ | Corchoionol C glucoside isomer 1 | 59/71/153/161/205/223 |
| 431 .1883 | 8 .13 | [M+CH_2_O_2_-H]^-^ | C_19_H_30_O_8_ | Corchoionol C glucoside isomer 2 | 59/71/153/161/205/223 |
| *Phenolic acids and derivatives* | | | | | |
| 153 .0190 | 3 .21 | [M-H]^-^ | C_7_H_6_O_4_ | Protocatechuic acid | 65/91/109 |
| 359 .0979 | 3 .48 | [M-H]^-^ | C_15_H_20_O_10_ | Glucosyringic acid isomer 1 | 95/123/138/153/182/197 |
| 353 .0863 | 3 .73 | [M-H]^-^ | C_16_H_18_O_9_ | Caffeoylquinic acid isomer 1 | 85/93/135/179/191 |
| 359 .0981 | 3 .81 | [M-H]^-^ | C_15_H_20_O_10_ | Glucosyringic acid isomer 2 | 95/123/138/153/197 |
| 353 .086 | 3 .96 | [M-H]^-^ | C_16_H_18_O_9_ | Caffeoylquinic acid isomer 2 | 85/93/135/179/191 |
| 285 .0611 | 4 .21 | [M-H]^-^ | C_12_H_14_O_8_ | Uralenneoside | 108/152 |
| 429 .1390 | 4 .48 | [M-H]^-^ | C_19_H_26_O_11_ | Bungeiside C | 59/89/135 |
| 137 .0241 | 5 .11 | [M-H]^-^ | C_7_H_6_O_3_ | Hydroxybenzoic acid | 65/93 |
| 353 .0864 | 6 .30 | [M-H]^-^ | C_16_H_18_O_9_ | Caffeoylquinic acid isomer 3 | 85/93/135/173/179/191 |
| 353 .0859 | 6 .69 | [M-H]^-^ | C_16_H_18_O_9_ | Caffeoylquinic acid isomer 4 | 85/93/135/173/179/191 |
| 353 .0860 | 6 .85 | [M-H]^-^ | C_16_H_18_O_9_ | Caffeoylquinic acid isomer 5 | 59/85/93/191 |
| 179 .0340 | 6 .89 | [M-H]^-^ | C_9_H_8_O_4_ | Caffeic acid | 65/89/108/135 |
| 367 .1015 | 6 .97 | [M-H]^-^ | C_17_H_20_O_9_ | Feruloylquinic acid isomer 1 | 93/134/193 |
| 135 .0450 | 7 .08 | [M-H]^-^ | C_8_H_8_O_2_ | Piceol | 65/92/120 |
| 371 .0973 | 7 .59 | [M-H]^-^ | C_16_H_20_O_10_ | Benzoyloxy-Hydroxypropoxy-trihydroxyoxane-carboxylic acid | 249/121/77 |
| 215 .0016 | 8 .44 | [M-H]^-^ | C_8_H_8_O_5_S | Acetylphenyl sulfate | 92/108/120/135 |
| 367 .1029 | 8 .45 | [M-H]^-^ | C_17_H_20_O_9_ | Feruloylquinic acid isomer 2 | 67/93/134/173/193 |
| 367 .1022 | 8 .74 | [M-H]^-^ | C_17_H_20_O_9_ | Feruloylquinic acid isomer 3 | 67/93/111/134/173 |
| 163 .0397 | 8 .96 | [M-H]^-^ | C_9_H_8_O_3_ | Coumaric acid | 65/93/119 |
| 515 .1171 | 10 .12 | [M-H]^-^ | C_25_H_24_O_12_ | Isochlorogenic acid isomer 1 | 93/135/173/179/191/353 |
| 515 .1145 | 10 .26 | [M-H]^-^ | C_25_H_24_O_12_ | Isochlorogenic acid isomer 2 | 93/135/173/179/191/353 |
| 515 .1175 | 10 .54 | [M-H]^-^ | C_25_H_24_O_12_ | Isochlorogenic acid isomer 3 | 93/135/173/179/191/353 |
| 515 .1191 | 10 .81 | [M-H]^-^ | C_25_H_24_O_12_ | Isochlorogenic acid isomer 4 | 135/173/191/353 |
| 207 .0658 | 11 .37 | [M-H]^-^ | C_11_H_12_O_4_ | Ethyl caffeate | 133/161/179 |
| *Flavonoids* | | | | | |
| 447 .1482 | 5 .43 | [M+CH_2_O_2_-H]^-^ | C_18_H_26_O_10_ | Icariside F2 isomer 1 | 59/71/89/101/131/161 |
| 447 .1479 | 5 .68 | [M+CH_2_O_2_-H]^-^ | C_18_H_26_O_10_ | Icariside F2 isomer 2 | 59/71/101/161/269/401 |
| 563 .1392 | 8 .61 | [M-H]^-^ | C_26_H_28_O_14_ | Vicenin isomer | 297/353/383/443/473 |
| 563 .1400 | 8 .86 | [M-H]^-^ | C_26_H_28_O_14_ | Vicenin isomer 2 | 297/353/383/443/473 |
| 270 .9912 | 9 .34 | [M-H]^-^ | C_10_H_8_O_7_S | Methoxy-oxochromenyl hydrogen sulfate | 104/120/148/176/191 |
| 593 .1469 | 9 .47 | [M-H]^-^ | C_27_H_30_O_15_ | Luteolin-rutinoside | 285 |
| 285 .0396 | 11 .28 | [M-H]^-^ | C_15_H_10_O_6_ | Luteolin | 65/107/135/151 |
| 287 .0549 | 11 .28 | [M-H]^-^ | C_15_H_12_O_6_ | Eriodictyol | 65/83/89/107/135/151 |
| 315 .0502 | 11 .93 | [M-H]^-^ | C_16_H_12_O_7_ | Methoxy-Luteolin | 65/133/136/300 |
| 329 .0661 | 12 .85 | [M-H]^-^ | C_17_H_14_O_7_ | Dimethoxy kaempferol | 65/271/299/314 |
| 299 .0552 | 12 .98 | [M-H]^-^ | C_16_H_12_O_6_ | Hispidulin | 65/117/137/256/284 |
| 343 .0815 | 13 .47 | [M-H]^-^ | C_18_H_16_O_7_ | Eupatilin | 65/270/285/298/313/328 |
| *Lignans* | | | | | |
| 583 .2007 | 7 .66 | [M+CH_2_O_2_-H]^-^ | C_26_H_34_O_12_ | Olivil Glucopyranoside derivate | 375/327/195/165/149 |
| 583 .2002 | 7 .85 | [M+CH_2_O_2_-H]^-^ | C_26_H_34_O_12_ | Olivil Glucopyranoside derivate | 375/327/195/165/149 |
| *Fatty acids and derivatives* |  |  |  |  |  |
| 197 .8090 | 1 .19 | [M-H]^-^ | C_9_H_18_O_10_S | Glyceryl sulfoquinovoside | 71/81/152/164/225 |
| 189 .0766 | 5 .10 | [M-H]^-^ | C_8_H_14_O_5_ | Carboxy hydroxy methyl hexanoate | 73/87/99/115/127 |
| 327 .2164 | 11 .93 | [M-H]^-^ | C_18_H_32_O_5_ | Trihydroxyoctadecadienoic acid | 55/69/85/18/211/229 |
| 329 .2322 | 12 .41 | [M-H]^-^ | C_18_H_34_O_5_ | Trihydroxyoctadecenoic acid | 211/171/139/99/57 |
| 293 .2112 | 15 .72 | [M-H]^-^ | C_18_H_30_O_3_ | Hydroxyoctadecatrienic acid | 59/71/121/183/221/232/275 |
| 309 .2058 | 16 .00 | [M-H]^-^ | C_18_H_30_O_4_ | Octadecatrienoic acid hydroperoxy | 291/195/119/71/59 |
| 313 .2374 | 17 .24 | [M-H]^-^ | C_18_H_34_O_4_ | Octadecanedioic acid | 295/269/251/183/155/125/71/59 |
| *Others* |  |  |  |  |  |
| 96 .9697 | 1 .15 | [M-H]^-^ | H_3_O_4_P | Phosphate | 62/78 |
| 96 .9610 | 1 .57 | [M-H]^-^ | H_2_SO_4_ | Sulfate | 64/80 |
| 447 .1490 | 7 .00 | [M+CH_2_O_2_-H]^-^ | C_18_H_26_O_10_ | Benzyl beta-primeveroside | 59/71/89/101/149/161/191/401 |
| 415 .1964 | 9 .52 | [M-H]^-^ | C_20_H_32_O_9_ | Ethylhydroxyjasmonate glucoside | 179 |

**Supporting information Table S2:** Occurrence of A. maritima phytochemicals in the extracts obtained with different solvents.

| **Proposed compound** | **Hexane** | **Ethyl acetate** | **Ethanol** | **Ethanol/Water** | **Water** |
| --- | --- | --- | --- | --- | --- |
| *Organic acids* |  |  |  |  |  |
| Hexa hydroxyheptanoic acid | - | + | + | + | + |
| Gluconic acid | - | - | + | + | + |
| Quinic acid | - | - | + | + | + |
| Malic acid | - | - | + | + | + |
| Citric acid | - | - | - | + | + |
| Aconitic acid | - | - | - | - | + |
| Isopropylmaleate isomer 1 | - | - | - | + | + |
| Isopropylmaleate isomer 2 | - | - | - | + | + |
| *Aminoacids and derivatives* |  |  |  |  |  |
| Glycosyl-asparagine | - | - | + | + | + |
| Tryptophan | - | - | - | + | + |
| *Sugars and Derivatives* |  |  |  |  |  |
| Sucrose | - | + | + | + | + |
| Fructose | - | + | + | + | + |
| Xylonic acid | - | - | + | + | + |
| Acetyl glucose | - | + | - | + | - |
| Heptose | - | - | - | - | + |
| Saccharide derivate | - | + | - | - | - |
| Daucic acid | - | - | - | + | + |
| Saccharide derivate | - | + | - | - | - |
| Xylobiose | - | + | - | - | - |
| Corchoionol C glucoside isomer 1 | - | - | - | + | + |
| Corchoionol C glucoside isomer 2 | - | - | - | + | - |
| *Phenolic acids and derivatives* |  |  |  |  |  |
| Protocatechuic acid | - | - | - | - | + |
| Glucosyringic acid isomer 1 | - | - | - | + | - |
| Caffeoylquinic acid isomer 1 | - | - | - | + | - |
| Glucosyringic acid isomer 2 | - | - | + | + | - |
| Caffeoylquinic acid isomer 2 | - | - | - | + | + |
| Uralenneoside | - | - | - | - | + |
| Bungeiside C | - | - | - | + | + |
| Hydroxybenzoic acid | - | - | - | + | + |
| Caffeoylquinic acid isomer 3 | - | - | - | + | + |
| Caffeoylquinic acid isomer 4 | - | - | - | + | - |
| Caffeoylquinic acid isomer 5 | - | - | + | + | + |
| Caffeic acid | - | - | - | - | + |
| Feruloylquinic acid isomer 1 | - | - | - | - | + |
| Piceol | - | + | + | + | + |
| Benzoyloxy-Hydroxypropoxy-trihydroxyoxane-carboxylic acid | - | - | - | + | + |
| Acetylphenyl sulfate | - | - | - | + | + |
| Feruloylquinic acid isomer 2 | - | - | - | - | + |
| Feruloylquinic acid isomer 3 | - | - | + | + | + |
| Coumaric acid | - | - | - | + | + |
| Isochlorogenic acid isomer 1 | - | + | + | + | + |
| Isochlorogenic acid isomer 2 | - | - | + | + | + |
| Isochlorogenic acid isomer 3 | - | - | + | + | + |
| Isochlorogenic acid isomer 4 | - | - | + | + | - |
| Ethyl caffeate | - | - | - | + | + |
| *Flavonoids* |  |  |  |  |  |
| Icariside F2 isomer 1 | - | - | - | + | - |
| Icariside F2 isomer 2 | - | - | - | + | + |
| Vicenin isomer | - | - | + | + | + |
| Vicenin isomer 2 | - | - | + | + | + |
| Methoxy-oxochromenyl hydrogen sulfate | - | - | - | + | + |
| Luteolin-rutinoside | - | - | + | + | - |
| Luteolin | - | + | + | + | - |
| Eriodictyol | - | + | + | + | - |
| Methoxy luteolin | - | + | + | + | - |
| Dimethoxy kaempferol | - | + | + | + | - |
| Hispidulin | - | + | + | + | + |
| Eupatilin | - | + | + | + | - |
| *Lignans* |  |  |  |  |  |
| Olivil Glucopyranoside derivate | - | - | - | + | + |
| Olivil Glucopyranoside derivate | - | - | - | + | + |
| *Fatty acids and derivatives* |  |  |  |  |  |
| Glyceryl sulfoquinovoside | - | - | + | + | + |
| Carboxy hydroxy methyl hexanoate | - | - | - | + | + |
| Trihydroxyoctadecadienoic acid | - | + | + | + | + |
| Trihydroxyoctadecenoic acid | - | + | + | + | + |
| Hydroxyoctadecatrienic acid | + | + | + | + | - |
| Octadecatrienoic acid hydroperoxy | + | - | + | - | - |
| Octadecanedioic acid | + | + | + | + | - |
| *Others* |  |  |  |  |  |
| Phosphate | + | + | - | + | + |
| Sulfate | - | - | - | + | + |
| Benzyl beta-primeveroside | - | - | - | + | + |
| Ethylhydroxyjasmonate glucoside | - | - | - | + | - |

**
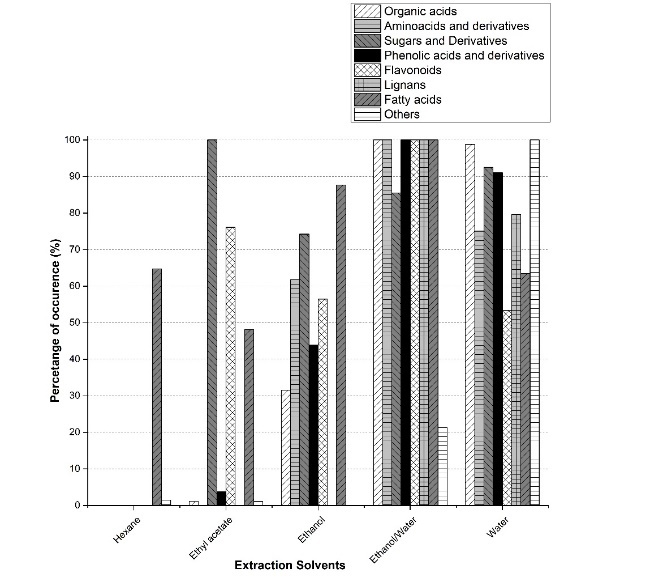
**

**Figure S1:** Percentage of phytochemical groups contribution to obtained extract according to extraction solvent used.
